# Coronavirus Disease 2019 (COVID-19) Pandemic Outburst: A Web-Based Cross-Sectional Study of the Knowledge, Attitudes, and Practices Among Undergraduate Students at a Tertiary Care Teaching Hospital in Tamil Nadu

**DOI:** 10.7759/cureus.81425

**Published:** 2025-03-29

**Authors:** Thakshana Vadivel, Kiran Belur, Kala Paneerselvam

**Affiliations:** 1 Pharmacology, SRM Medical College Hospital and Research Centre, Chengalpattu, IND

**Keywords:** adolescents, behavior, covid-19, infection, prevention, spread

## Abstract

Background: The novel coronavirus disease 2019 (COVID-19) is a highly transmissible infection caused by the severe acute respiratory syndrome coronavirus 2 (SARS-CoV-2) virus. The World Health Organization (WHO) affirmed COVID-19 as a global pandemic on March 11, 2020. During the first wave of the pandemic, >150 million children and adolescents in 165 countries were impacted. A major concern of the WHO was to rapidly identify the cases, perform the diagnostic tests, and conduct regular follow-ups to prevent further transmission. The objective of the present web-based survey was to assess the knowledge, attitudes, and practices (KAP) related to COVID-19 among undergraduate students at a tertiary care teaching hospital in Tamil Nadu during the third wave and to compare KAP levels between medical and non-medical students, as well as evaluate gender-based differences.

Methodology: The present web-based cross-sectional study was conducted among medical and other allied health sciences undergraduate students studying at SRM Medical College Hospital and Research Centre, Chengalpattu, India. A dichotomous questionnaire containing 24 questions was validated, and written informed consent was obtained from all the study participants. The study was carried out in the last week of January 2022 during the third wave of the pandemic. The present cross-sectional survey showed that basic questions regarding KAP related to the COVID-19 infection were included to test the undergraduates' opinion about the protocol. Study participants were selected using a simple random sampling method, and the required sample size for this study was 631.

Results: Out of 728 participants, 343 (47.11%) were males and 385 (52.88%) were females. Of the total study participants, 358 (49.17%) were medical undergraduates, while 370 (50.82%) were selected from other allied health sciences, categorized as non-medical students. In the current study, 5273 (80.48%) of the participant responses indicated a substantial understanding about the COVID-19 infection. In the attitude section, 5466 (83.48%) of the accurate replies from the survey participants indicated a favorable disposition towards the highly infectious disease. The practice part indicated that 4158 (95.19%) of the responses from the study population demonstrated commendable practices for the COVID-19 infection. The study emphasizes that the public strictly adhere to the COVID-19 prevention protocol to control the spread of the infection.

Conclusion: It is crucial to emphasize to the undergraduate students studying at a tertiary care teaching hospital about the importance of vaccination against COVID-19 and booster doses to keep the infection under control. Caution should be taken against new variants of SARS-CoV-2 and their mode of transmission. Viral genomic sequencing and treatment-resistant strains might endanger the global public health in the future, and it is essential to keep a check on the virulent strains at regular intervals to protect the nation against COVID-19.

## Introduction

The first case of pneumonia of unknown aetiology was reported from Wuhan, China, in December 2019 [1]. The main cause of the highly contagious infection is the severe acute respiratory syndrome coronavirus 2 (SARS-CoV-2) virus which is popularly called the novel coronavirus disease 2019 (COVID-19) [1]. The World Health Organization (WHO) declared COVID-19 as a global pandemic on March 11, 2020 [2]. A new era of COVID-19 started with the rise in the number of cases from March 2020. During the first wave of the pandemic, >150 million children and adolescents in 165 countries were impacted [3]. The name became widespread in every household, and life came to a sudden standstill. COVID-19 affected every nation without showing any discrimination, from toddlers to the elderly, the poor to the rich, and the uneducated to the educated, and it spread across age, gender, caste, creed, religion, and region. The major concern of the WHO was to rapidly identify the cases, perform the diagnostic tests, and conduct regular follow-ups to prevent further transmission. It was a wake-up call for all the nations. In India, the government declared a nationwide total lockdown to prevent the rapid spread of transmission. International travel was banned, and all shops, educational institutions, and transport services were closed. People were educated to wisely avoid unnecessary crowding and encouraged to wear masks, follow handwashing with soap and water, carry sanitiser, and maintain a social distance of 6 m. Globally, COVID-19 caused 760,360,956 confirmed cases and 6,873,477 confirmed deaths, and vaccine doses were administered to 13,232,780,775 as of March 16, 2023 [4]. It has evolved as the most devastating health threat after the influenza pandemic in 1918. People were advised to look for the symptoms which appear 2-14 days after exposure to the virus. It included fever or chills, sore throat, cough, new loss of taste or smell, congestion or runny nose, nausea or vomiting, diarrhoea, shortness of breath or difficulty in breathing, fatigue, body aches, and headache. Immediate hospitalization was done for patients with reduced oxygen saturation levels, and severe respiratory distress was mostly seen in patients with cardiac ailments and diabetes and elderly patients. The proverb "Prevention is better than cure" is apt to this pandemic [5]. The primary goal in stopping the transmission of the infection is to educate people with the correct data. The whole country was on high alert with plenty of information through different mass communications like television, newspapers, WhatsApp, Facebook, Instagram, and YouTube. The Aarogya Setu mobile application (National Informatics Centre Services Inc. (NIC), New Delhi, India) was established by the Government of India to enlighten the people about the possibilities of getting the infection and the preventive measures and appropriate advisories associated with COVID-19. Even the ringtone of telephones was changed to a 30-second audio clip raising awareness about COVID-19. The primary medium of controlling the infection and combating the spread of the pandemic was to disseminate information about it through roads, walls, common places, banners, movies, and audio materials. In the era of infodemic, behavior change communication really changed the attitudes or practices in the community. In the management of infectious illnesses, practical application supersedes theoretical understanding. This study can demonstrate the efficacy with which students adhere to mask-wearing, hand hygiene, and physical distancing protocols. COVID-19 proficiency may differ across students despite extensive healthcare education. Altering these mindsets can enhance teamwork and proactivity in pandemic management. Medical and healthcare students constitute the future workforce of any nation. They must prepare for pandemics and health emergencies. A global health issue is impacting the practices of these future healthcare professionals, and this study will elucidate the extent of that influence. Their training, attitudes, and behaviors will influence their capacity to manage future pandemic crises. The objective of the present web-based survey was to assess the knowledge, attitudes, and practices (KAP) related to COVID-19 among undergraduate students at a tertiary care teaching hospital in Tamil Nadu during the third wave and to compare KAP levels between medical and non-medical students, as well as evaluate gender-based differences.

## Materials and methods

Study design and setting

The current web-based cross-sectional survey was carried out to assess the KAP of undergraduate students studying medicine and other allied health sciences at SRM Medical College Hospital and Research Centre, Chengalpattu, India. The study was conducted for two months, starting in the third week of January 2022, during the third wave of the COVID-19 pandemic. The study was started with consent from the Institutional Ethics Committee of SRM Medical College Hospital and Research Centre. Every study participant who agreed to take part in the research provided signed informed consent.

Study participants

Undergraduate students of both genders who were enrolled at SRM Medical College Hospital and Research Centre and above the age of 18 were included in the study after providing written informed consent and consenting to participate. The study did not include those who were unwilling to participate.

Study instrument

The questionnaire consisted of 24 dichotomous (Yes/No) questions, categorized into three domains: knowledge (nine questions) which covered symptoms, modes of transmission, and preventive measures of COVID-19; attitude (nine questions) which assessed perceptions, confidence in guidelines, and willingness to adhere to preventive measures; and practice (six questions) which evaluated mask usage, hand hygiene, and social distancing practices. Content validity was assessed by a panel of three experts in pharmacology, infectious diseases, and epidemiology. Modifications were made based on expert feedback to enhance clarity, relevance, and comprehension. The internal consistency of the questionnaire was evaluated using Cronbach's alpha, which yielded an acceptable reliability score of 0.78. A pilot study was conducted with 60 participants (30 medical and 30 allied health students) to evaluate question clarity, response time, and overall usability. Participants were selected randomly from the student database using Microsoft Excel's random number generator (Microsoft Corp., Redmond, WA, USA). Feedback was collected on the ambiguity, difficulty, and relevance of the questions.

Study procedure

The study commenced in the third week of January 2022 following permission from the Institutional Ethics Committee of SRM Medical College Hospital and Research Centre (approval number: 3015A/IEC/2021). Study participants were selected using a simple random sampling method. The study commenced after obtaining signed informed consent from each of the study participants. A total of 728 undergraduate students, comprising both male and female individuals aged 18 years and older, engaged in medical or allied health sciences programs at the SRM Medical College Hospital and Research Centre, Chengalpattu, India, were included in the study. The study methodology was comprehensively elucidated to the selected participants via simple random sampling regarding the significance of understanding symptoms and mode of transmission and exercising caution to prevent the spread of the deadly viral infection. Demographic information, including age, gender, and course, was collected using a proforma. The COVID-19 assessment comprised three sections: knowledge (nine questions), attitude (nine questions), and practice (six questions). The poll consisted entirely of closed-ended questions. This study utilized a standardized scoring methodology based on a questionnaire. In the KAP components of the two-point scale, a score of 1 was assigned for an affirmative response and 0 for a negative response. The knowledge score ranged from 0 to 9, the attitude score ranged from 0 to 9, the practice score ranged from 0 to 6, and the overall score ranged from 0 to 24. Students disseminated the questionnaire through Google Forms (Google LLC, Mountain View, CA, USA).

Sample size calculation

The sample size was calculated using the following formula: $n = \frac{Z^2 \cdot p \cdot (1 - p)}{d^2}$. Here, $Z = 1.96$ is the Z-value corresponding to a 95% confidence level ($\alpha = 0.05$ for a two-sided test), $p = 0.82$ is the expected proportion in the population, $(1 - p = 0.18)$ is the complement of the proportion, and $d = 0.03$ is the desired absolute precision (margin of error). Thus, the required sample size for this study is $n = 631$ [6]. To account for a 10% attrition rate (non-responses/dropouts), the sample size was adjusted to 702. To ensure adequate representation, a total of 728 students were included in the study.

Statistical analysis

Data were analyzed using the Social Science Statistics online software. The chi-squared test was utilized to compare the differences between genders and medical and non-medical students. P<0.001 was considered statistically significant.

## Results

**Figure 1:**
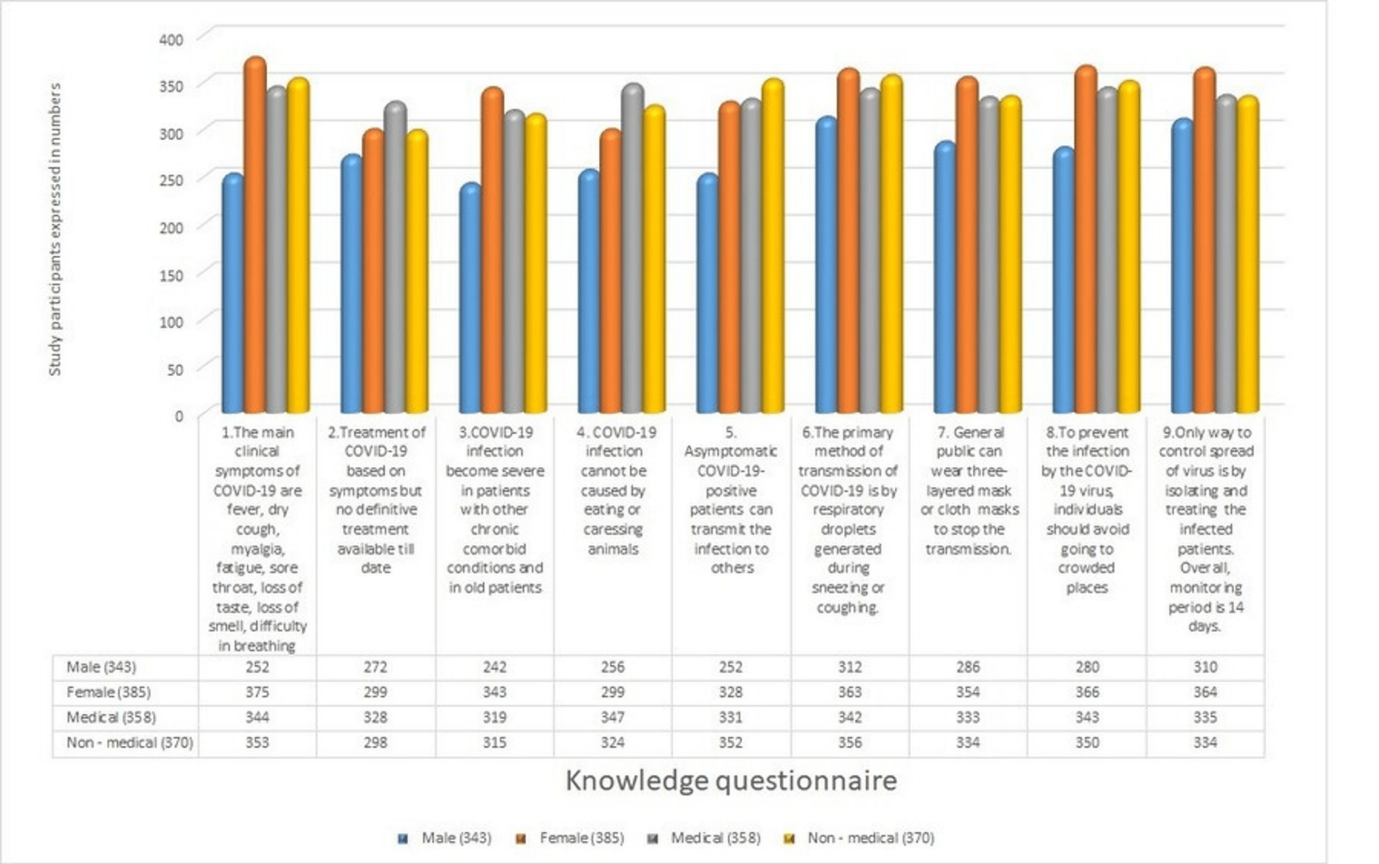
COVID-19 knowledge questionnaire for undergraduate students COVID-19: coronavirus disease 2019

In this study, of the 728 participants, 343 (47.11%) were male and 385 (52.88%) were female. Of the total study participants, 358 (49.17%) were medical undergraduates, while 370 (50.82%) were students from other allied health sciences, categorized as non-medical students. The average age of the students was 19.41. In the current study, 252 males (73.46%) out of 343 and 375 females (97.40%) out of 385 exhibited knowledge of the signs of viral infection. Medical students showed a superior understanding of symptoms, with 344 (96.09%) indicating expertise, in contrast to non-medical students. No definitive treatment for COVID-19 exists; only symptomatic management is reported by 328 (91.62%) medical students and 298 (80.54%) non-medical students. Gender differences in responses to this question were minimal. Co-morbid conditions and the elderly exhibit increased susceptibility to COVID-19, as indicated by responses from 343 (89.09%) female participants compared to 242 (70.55%) male participants. The transmission of the infection does not occur through the consumption or handling of animals, precisely identified by 347 (96.92%) medical students. Asymptomatic COVID-19-positive individuals may pass on the virus, as indicated by 352 (95.13%) non-medical students, in contrast with 331 (92.45%) medical students. Respiratory droplets released during sneezing and coughing represent the most common mode of transmission, as shown by 342 (95.53%) medical students and 356 (96.21%) non-medical students. The general population can protect themselves by utilizing a three-layered mask or cloth mask, as accurately identified by 333 (93.01%) medical undergraduates. Participants largely concurred that individuals should avoid crowded places, with the exception of males, among whom only 280 (81.63%) expressed agreement with this statement. The isolation of COVID-19-positive patients is the most successful strategy for controlling the spread of this infection, as concurred by the majority of the study participants (Figure 1). 

**Figure 2:**
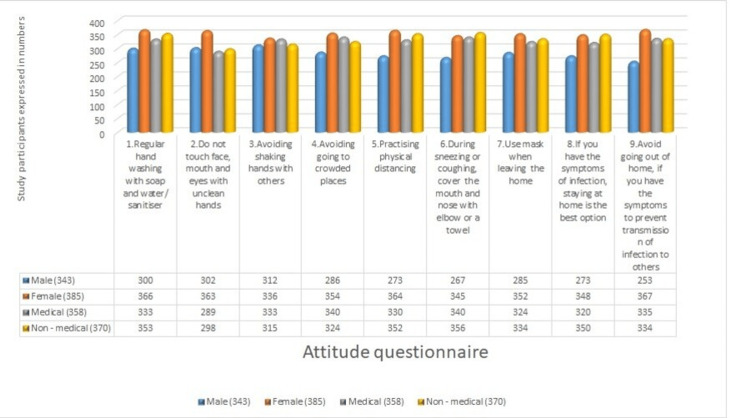
COVID-19 attitude questionnaire for undergraduate students COVID-19: coronavirus disease 2019

In the attitude section, regular handwashing is regarded as the main approach for combating the spread, as acknowledged by 366 (95.06%) females and 353 (95.40%) non-medical students. Among the female participants, 363 (94.28%) admitted that touching the face, mouth, and eyes with unclean hands is a contributing cause to transmission. Avoidance of shaking hands may contribute to the spread, as indicated by 333 (93.01%) medical students and 312 (90.96%) male subjects. A total of 340 medical students, representing 94.97%, reported avoiding crowded places. Physical distancing practices were adhered to by 352 (95.13%) non-medical students, in contrast to 330 (92.17%) medical students. Among non-medical students, 356 individuals (96.12%) practiced covering their mouths during sneezing and coughing. The majority of the participants adhered to mask-wearing when leaving their homes, as indicated by our study. Among symptomatic patients, 350 (94.59%) non-medical students accepted the recommendation to remain at home to prevent further transmission. A majority of female students, 367 (95.32%), reported avoiding going out of the home when experiencing symptoms (Figure 2).

**Figure 3:**
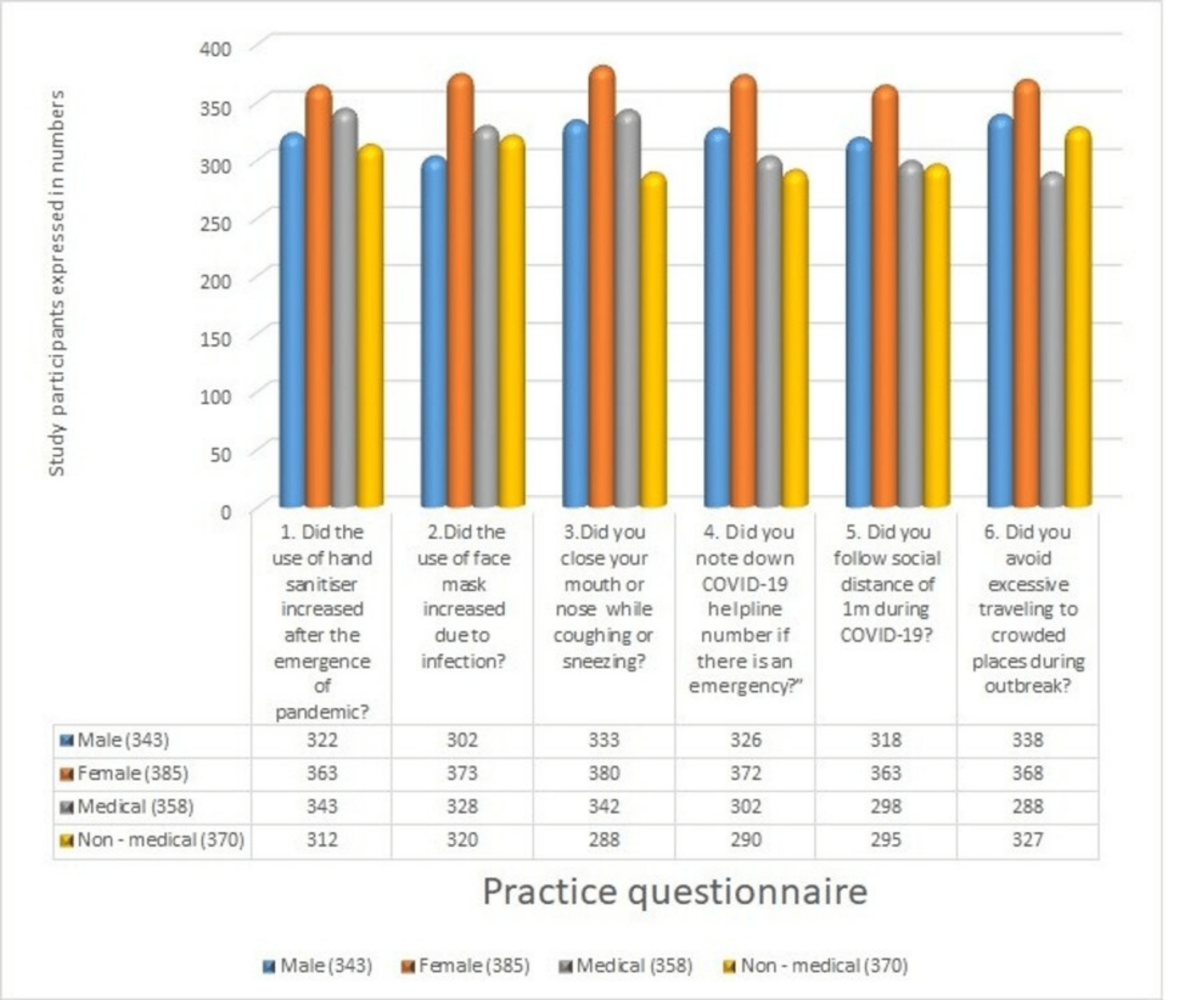
COVID-19 practice questionnaire for undergraduate students COVID-19: coronavirus disease 2019

In the practice section, the use of hand sanitiser increased following the emergence of the pandemic, as reported by 343 (95.81%) medical students, with 363 (94.28%) being female and 322 (93.88%) being male participants. The current study reported an increase in face mask usage among 373 (96.88%) female participants. The COVID-19 pandemic has led to an increase in the practice of covering the mouth and nose during coughing and sneezing, observed in 333 (97.09%) males and 380 (98.70%) females. Emergency numbers for COVID-19 were recorded by 363 (94.28%) female participants and 318 (92.71%) male participants in the study. Avoidance of unnecessary visits to crowded places was reported by 338 (98.54%) male participants and 368 (95.58%) female participants. In the current study, 327 (88.37%) non-medical students and 288 (80.44%) medical students refrained from unnecessary trips (Figure 3). 

A comparative analysis of knowledge regarding COVID-19 between male and female participants, as well as between medical and non-medical students, indicated a statistically significant greater awareness among female participants. This included knowledge of clinical symptoms, co-morbid conditions, the increased severity of COVID-19 in the elderly, and the importance of avoiding crowded places to prevent transmission (p<0.001). A statistically significant difference exists between medical and non-medical students regarding awareness of the absence of a definitive treatment for the COVID-19 infection and the understanding that infection is not transmitted through consumption or touching animals (Table 1).

**Table 1:** Comparison of COVID-19 knowledge among different groups The chi-squared test was used to compare the categorical data. * represents a p-value of <0.001 which is statistically significant N: frequency; %: percentage; COVID-19: coronavirus disease 2019

	Gender-wise difference						Course-wise difference					
Knowledge questions	Male (N=343)	%	Female (N=385)	%	χ²	p	Medical (N=358)	%	Non-medical (N=370)	%	χ²	p
K1 correct	252	73.46	375	97.40	86.95	<0.001*	344	96.089	353	95.40	0.208	0.647
K2 correct	272	79.30	299	77.66	0.287	0.591	328	91.62	298	80.54	18.539	<0.001*
K3 correct	242	70.55	343	89.09	39.48	<0.001*	319	89.10	315	85.13	2.551	0.110
K4 correct	256	74.63	299	77.66	0.91	0.338	347	96.92	324	87.56	22.087	<0.001*
K5 correct	252	73.46	328	85.19	15.39	0.001*	331	92.45	352	95.13	2.248	0.133
K6 correct	312	90.96	363	94.28	2.96	0.084	342	95.53	356	96.21	0.216	0.641
K7 correct	286	83.38	354	91.94	12.52	<0.001*	333	93.01	334	90.27	1.787	0.181
K8 correct	280	81.63	366	95.06	32.74	<0.001*	343	95.81	350	94.59	0.587	0.443
K9 correct	310	90.37	364	94.54	4.58	0.032	335	93.57	334	90.27	2.668	0.1023

A similar comparison in the attitude section suggested that medical students exhibited a more favorable attitude towards refraining from touching the face, mouth, and eyes with unclean hands, as well as advising symptomatic patients to avoid leaving home and wear a mask when leaving home, compared to non-medical students. This difference was statistically significant. Female participants exhibited a statistically significant more favorable attitude than male participants regarding the practices of regular handwashing, avoiding going to crowded places, physical distancing, using a mask when going out, covering the mouth and nose during coughing and sneezing, and refraining from going out while experiencing COVID-19 symptoms (Table 2). 

**Table 2:** Comparison of COVID-19 attitudes among different groups The chi-squared test was used to compare the categorical data. * represents a p-value of <0.001 which is statistically significant N: frequency; %: percentage; COVID-19: coronavirus disease 2019

	Gender-wise difference						Course-wise difference					
Attitude questions	Male (N=343)	%	Female (N=385)	%	χ²	p	Medical (N=358)	%	Non-medical (N=370)	%	χ²	p
A1 correct	300	87.464	366	95.06	13.452	0.001*	333	93.01	342	92.43	2.14	0.142
A2 correct	302	88.04	363	94.28	8.932	0.002	289	80.72	247	66.75	18.28	0.001*
A3 correct	312	90.96	336	87.27	2.52	0.11	333	93.01	327	88.37	4.62	0.03
A4 correct	286	83.38	354	91.94	12.52		340	94.97	355	95.94	0.398	0.52
A5 correct	273	79.59	364	94.54	37.08	<0.001*	330	92.17	345	93.24	0.305	0.580
A6 correct	267	77.84	345	89.61	45.591	<0.001*	340	94.97	352	95.13	0.010	0.919
A7 correct	285	83.09	352	91.42	11.530	<0.001*	324	90.50	304	82.16	10.682	0.001*
A8 correct	273	79.59	348	90.39	16.868	<0.001*	320	89.38	325	87.83	0.4314	0.511
A9 correct	253	73.76	367	95.32	66.76	<0.001*	335	93.57	298	80.54	27.24	<0.001*

In a comparative analysis of practice among different groups, it was reported that medical students exhibited a greater increase in the use of hand sanitiser and a better-developed habit of covering their nose and mouth when coughing and sneezing during the pandemic, compared to non-medical students, with a statistically significant difference observed. The usage of face masks was significantly higher among female participants than male participants (Table 3).

**Table 3:** Comparison of COVID-19 practices among different groups The chi-squared test was used to compare the categorical data. * represents a p-value of <0.001 which is statistically significant N: frequency; %: percentage; COVID-19: coronavirus disease 2019

	Gender-wise difference						Course-wise difference					
Practice questions	Male (N=343)	%	Female (N=385)	%	X^2^	p	Medical (N=358)	%	Non-medical (N=370)	%	X^2^	p
P1 correct	322	93.878	363	94.28	0.054	0.81	343	95.81	312	84.32	26.605	<0.001*
P2 correct	302	88.047	373	96.88	20.98	<0.001*	328	91.62	320	86.48	4.902	0.026
P3 correct	333	97.085	380	98.70	2.349	0.12	342	95.53	288	77.83	48.893	<0.001*
P4 correct	326	95.044	372	96.62	1.145	0.28	302	84.35	290	78.37	4.281	0.038
P5 correct	318	92.711	363	94.28	0.744	0.388	298	83.24	295	79.73	1.484	0.223
P6 correct	338	98.542	368	95.58	5.415	0.01	288	80.44	327	88.37	8.729	0.003

## Discussion

Knowledge section

This study found that 5273 (80.48%) of the questions were answered correctly by participants, indicating a significant level of knowledge about the COVID-19 pandemic. Among the knowledge questionnaire, female students had statistically significant knowledge about COVID-19, except for the three statements: treatment of COVID-19 based on symptoms but no definitive treatment available to date, eating or contacting wild animals would not result in the infection by the COVID-19 virus, and the main mode of transmission of the COVID-19 infection is by respiratory droplets while they are sneezing or coughing. All other statements were correctly answered by female students compared to male students. These exceptions suggest that certain aspects of COVID-19 knowledge were either misunderstood or not emphasized enough in public health messaging. In addition, medical students performed better compared to non-medical students in answering statements such as eating or contacting wild animals would not result in infection by the COVID-19 virus and the COVID-19 virus spreads via respiratory droplets of infected individuals. The other six statements in the knowledge section showed no difference between medical and non-medical students. This could be attributed to their academic background in epidemiology and infectious disease transmission. Non-medical students, on the other hand, may have relied primarily on general public health information, which may not have covered these specific details comprehensively. A study conducted in Jordan did not show a significant difference in the knowledge regarding the COVID-19 infection among medical and non-medical students [7]. A Saudi Arabian study conducted among the public showed that 2797 (81.64%) had good knowledge about COVID-19, and results were in accordance with the present study findings [8].

Attitude section

In the attitude section, 2860 (83.48%) of the responses from study participants indicated a positive attitude towards highly infectious diseases. In this section, female students had better knowledge in answering all the questions compared to male students except for the following statement: avoiding shaking hands with others. Medical students answered all the questions better than non-medical students except for answering the following statements: avoiding touching the face with unwashed hands, avoiding shaking hands with others, using a mask when leaving home, and avoiding going out of the home if the person had the symptoms to prevent the transmission of infection to others. Medical students' statistically significant improvement in answering questions related to unnecessary traveling and increased mask usage during the COVID-19 outbreak is likely a result of their medical training, which emphasizes preventive health practices and public health measures compared to non-medical students. This difference in attitude suggests that medical education not only provides technical knowledge but also instills attitudes that prioritize preventive health behaviors, which may contribute to medical students serving as informal health educators within their communities during health crises. Notably, the domains in which medical students failed to surpass their non-medical peers in responding to inquiries regarding face touching, handshaking, mask usage upon leaving home, and self-isolation during symptomatic periods pertain to fundamental hygiene practices that were extensively highlighted in public communications. This finding implies that well-designed public health campaigns can successfully instill positive health attitudes across educational backgrounds when messages are simple, actionable, and consistently reinforced. A cross-sectional study conducted in Uttarakhand among medical students showed a slightly higher percentage of knowledge (328 (92.7%)), but the attitude percentage was reported to be similar to the current study findings (283 (80%)) [9]. A national online cross-sectional survey indicated that the Indian population exhibited good knowledge (293 (58.6%)) and showed positive attitudes (311 (62.1%)) towards the COVID-19 infection [10]. The discrepancy between higher knowledge levels and relatively lower positive attitudes observed in multiple studies suggests that knowledge alone may not be sufficient to drive attitudinal change, pointing to the need for public health interventions that address emotional and psychological barriers to adopting preventive behaviors.

Practice section

The current study disclosed that the practice section indicated 4158 (95.19%) of the questions were answered accurately by the study population, confirming effective practices regarding the COVID-19 infection. Females in the current study demonstrated the best practices in dealing with the COVID-19 infection by properly answering every question on a practice questionnaire, except for two, namely, (1) "Did the use of face masks increase as a result of illness?" and (2) "Did you observe the one-meter social distance during the COVID-19 pandemic?", in contrast to participants who were male. The notably high practice score compared to knowledge and attitude scores across the study population is particularly significant, as it indicates that participants were implementing preventive behaviors even in cases where their understanding or attitudes might have been less than optimal. This pattern suggests that during public health emergencies, behavioral compliance may be driven by factors beyond just knowledge and attitudes, such as social norms, peer influence, mandatory policies, or fear of consequences. Medical students showed statistically significant improvement in answering questions like "Did you avoid unnecessary travel or outings during the outbreak?" and "Did the outbreak of the COVID-19 virus make you wear the mask more often than you used to?" compared to non-medical students. In this case, female students may have more strongly internalized COVID-19 safety behaviors, leading to a higher rate of compliance with recommendations such as mask-wearing and maintaining social distance. The gender difference in compliance with safety measures could be attributed to several factors, including differences in risk perception and a greater tendency towards health-protective behaviors among females. An Iranian study showed that the knowledge score regarding COVID-19 was 5223 (60.8%) among the general population, revealing moderate knowledge towards the disease. The same study showed 7731 (90%) and 7645 (89%) in the attitude and practice sections, which were in association with the present study results [11]. The significantly higher attitude and practice scores compared to knowledge scores in the Iranian study mirror our findings, reinforcing the observation that during public health emergencies, people may adopt recommended behaviors even without a complete understanding of the underlying science. A study conducted in Jammu and Kashmir observed a total knowledge score of 88.9%, a positive attitude among 73.3%, and that 93% had positive practices regarding the COVID-19 infection [12]. This regional variation within India highlights the potential influence of local factors, including differential exposure to public health messaging, regional policy implementation, and community-level social norms. One more cross-sectional study conducted in higher educational institutions across 22 states in India presented that 820 (65.5%) students had good knowledge about the disease. A positive attitude towards COVID-19 was seen in 888 (71%) of them; 835 (66.7%) followed better practices to alleviate the COVID-19 infection [13]. This nationwide study provides a valuable benchmark for comparison, suggesting that our study population demonstrated above-average KAP compared to the broader Indian student population, which could reflect regional differences in educational quality, healthcare access, or public health communication effectiveness.

Overall KAP results

In the present study, there were 6552 knowledge-related questions, 6552 attitude-relevant questions, and 4368 practice-related questions pertaining to COVID-19. The overall KAP score of the present study was remarkable indicating that 4158 (95.19%) of the participant replies adhered to optimal COVID-19 prevention methods, 5273 (80.48%) of the responses exhibited sufficient knowledge, and 5466 (83.48%) of the accurate responses indicated a favorable attitude among participants. Considering all KAP results, female students' overall attitudes towards COVID-19 questions were statistically significant, but there were no statistically significant results seen in the knowledge and practice sections among all students. The overall KAP results also showed statistically significant differences between medical students and non-medical students; however, it reported no statistically significant difference between the two genders. The complex pattern of gender differences, significant in attitudes but not in overall knowledge or practice, suggests nuanced gender-based variations in how health information is processed and internalized. Females may be more receptive to the emotional and social aspects of health messaging, leading to stronger attitudinal responses, while practical behaviors may be more uniformly adopted across genders due to institutional requirements or societal pressures. The present study showed that female students outperformed the male students in the knowledge regarding COVID-19, except for the questions as follows: "There is currently no effective cure for COVID-19, but early symptomatic and supportive treatment can help most patients recover from the infection", "Eating or contacting wild animals would not result in the infection by the COVID-19 virus", and "The COVID-19 virus spreads via respiratory droplets of infected individuals". As of today, there is no definitive treatment for COVID-19, and treatment is given depending on the symptomatology. Paracetamol, antibiotics to treat respiratory tract infection, glucocorticoids, and other drugs having anti-inflammatory and anti-viral properties were tried in COVID-19-positive patients. There is no proof in the scientific literature that the COVID-19 infection was caused by eating or caressing animals. It is well known that COVID-19 spreads through respiratory droplets; the WHO made it mandatory to wear masks by all individuals to prevent the spread of the infection. The specific knowledge gaps identified across both genders regarding treatment options and transmission mechanisms represent critical areas for improved health communication. The misconceptions about treatment efficacy could potentially lead to inappropriate self-medication or false security, while misunderstandings about transmission routes might compromise adherence to critical preventive measures. These findings highlight the importance of focusing public health education on correcting these specific misconceptions. The students were less knowledgeable about respiratory droplets, which were the main mode of transmission of COVID-19. However, study participants were aware that an asymptomatic COVID-19-positive patient is able to transmit the infection, which was in association with the cluster outbreak study conducted in Shaanxi Province, China [14]. This awareness of asymptomatic transmission represents an important public health success, as this concept was initially counterintuitive to many people's understanding of disease spread yet became widely recognized among the study population. In a similar study, 52.4% and 44.8% of the individuals displayed positive attitudes and behaviors, respectively, but only 33% of the participants demonstrated strong knowledge, indicating that the total KAP was low among the Bangladeshi community [15]. The stark contrast between our findings and the Bangladeshi study highlights the potential influence of socioeconomic factors, healthcare infrastructure, and education systems on pandemic knowledge and behaviors. The current study found that 342 (95.53%) medical students and 356 (96.21%) non-medical students knew about the COVID-19's mode of transmission, which is closely related to 596 (94.5%) medical students who participated in a descriptive cross-sectional study in Mangalore. In the Mangalore study, 464 (73.6%) participants demonstrated a lower understanding of three-layered masks compared to 333 (93.01%) medical students and 334 (90.27%) non-medical students, who exhibited a comparatively higher comprehension, as indicated by the current study findings [16]. The higher rates of knowledge regarding mask usage in our study compared to the Mangalore study could reflect temporal differences in data collection, as mask-related public health messaging evolved significantly throughout the pandemic. Alternatively, it might indicate regional variations in the emphasis placed on different preventive measures by local health authorities or media. In the current study, 343 (95.81%) medical students and 350 (94.59%) non-medical students refrain from crowded areas to mitigate infection spread. In contrast, a cross-sectional study conducted in the National Capital Territory of Delhi and the National Capital Region (NCR) indicated that 750 (74.85%) individuals exhibited lesser awareness regarding the avoidance of crowded places during COVID-19 [17]. This disparity between student populations and the general public highlights the potential influence of educational attainment on health behavior adoption, suggesting that higher education may facilitate better comprehension and implementation of public health guidelines. In the current survey, it was noted that the overall knowledge component was 5273 (80.48%), with 320 (89.38%) medical students and 350 (94.59%) non-medical students believing that remaining at home is the optimal strategy to mitigate further disease transmission. In 2020, a community-based prospective cross-sectional research of rural residents in Sidama regional state, Southern Ethiopia, revealed that COVID-19 KAP scores were 90%, 82.4%, and 65%, respectively [18]. The notably lower practice score in the Ethiopian study compared to our findings may reflect structural barriers to implementing preventive behaviors in rural settings, such as limited access to personal protective equipment and water for handwashing or economic constraints preventing work-from-home compliance. This comparison emphasizes the importance of addressing practical barriers to behavior change in addition to knowledge and attitude factors. According to a mixed study design (cross-sectional and systematic review), the prevalence of good knowledge, positive attitude, and frequent practice regarding the COVID-19 pandemic was 388 (52.6%), 384 (51.8%), and 424 (57.1%), respectively, in Southeast and South Asia [19], which is in contrast with our study findings. The substantially higher KAP scores in our study compared to this regional average suggest that our study population may represent a particularly well-informed and compliant subset, possibly due to factors such as higher educational status, better access to health information, or more effective local public health initiatives. One more cross-sectional study conducted in India among the general population also noted that 1047 (81%) of the participants had good knowledge, 995 (77%) had better attitude, and 1079 (83.5%) followed good practices regarding the pandemic which would have been a vital in controlling the spread of the infection [20]. The close alignment of these findings with our results provides further validation for our conclusions and suggests the potential emergence of consistent patterns in COVID-19 KAP across diverse Indian populations. This consistency could indicate the effectiveness of national-level public health communication strategies in reaching diverse demographic groups.

Strengths of the study

A KAP study on COVID-19 among undergraduate students at a tertiary care teaching hospital can provide valuable insights into how well students understand the virus, the attitudes they hold towards it, and their preventive behaviors. This group is at a transitional phase of life and may have different health perceptions compared to other demographics. Studying their KAP related to COVID-19 could inform how to better communicate health messages to younger adults. Comparing the responses of male and female students could highlight potential differences in their understanding of COVID-19, health behaviors, or risk perception, revealing if gender influences how individuals respond to health crises. Comparing medical with non-medical students can help assess if their professional training influences their KAP regarding the pandemic. It could reveal whether medical training provides a clearer understanding of scientific information and leads to more responsible health practices.

Limitations of the study

The present study offers a valuable insight into students' KAP at a single point in time, but has limited ability to demonstrate cause-effect relation; it does not provide insights into how students' KAP change over time. The smaller sample size of this study may not be representative of the broader student population. Students may provide answers that they believe are socially acceptable or expected, rather than their true opinions or behaviors. The study uses an online survey; it could exclude students who have limited access to the internet, which might cause bias. Study participants may not accurately recall their past behaviors or experiences, leading to inaccuracies in the data. Future research should implement qualitative interviews in addition to surveys or longitudinal studies to monitor changes over time which could help researchers get contextual elements of the study.

## Conclusions

The study demonstrated that participants possessed a strong understanding of the COVID-19 pandemic, with 5,273 (80.48%) of the knowledge-related questions answered correctly, while 2,860 (83.48%) of the responses reflected a positive attitude towards highly infectious diseases, and 4,158 (95.19%) of the practice-related questions were answered accurately, indicating excellent adherence to COVID-19 preventive measures. This study provides valuable insights into the KAP of undergraduate medical and allied health sciences students regarding COVID-19 at a tertiary care teaching hospital in Tamil Nadu. The findings highlight the level of awareness, perceptions, and adherence to preventive measures among future healthcare professionals during the pandemic. The present cross-sectional survey showed that simple problems related to KAP about COVID-19 were included to test undergraduate students' opinions about the protocol. The findings indicate that while participants demonstrated good overall knowledge and adherence to preventive practices, notable differences were observed across gender and academic disciplines. Medical students and female participants tended to perform better in KAP domains, highlighting the influence of formal medical education and possible gender-related differences in health awareness and risk perception. Given the critical role of healthcare students in pandemic response and future public health crises, targeted educational interventions and training programs are essential to bridge any gaps in attitudes and practices. Strengthening awareness campaigns and incorporating pandemic preparedness into medical curricula could further enhance students' ability to respond effectively to similar health emergencies in the future. By providing an evidence-based understanding of students' KAP towards COVID-19, this study contributes to the ongoing efforts to enhance public health preparedness and infection control measures in healthcare settings.
